# Animal Welfare and the United Nations Sustainable Development Goals

**DOI:** 10.3389/fvets.2019.00336

**Published:** 2019-10-10

**Authors:** Linda Keeling, Håkan Tunón, Gabriela Olmos Antillón, Charlotte Berg, Mike Jones, Leopoldo Stuardo, Janice Swanson, Anna Wallenbeck, Christoph Winckler, Harry Blokhuis

**Affiliations:** ^1^Department of Animal Environment and Health, Swedish University of Agricultural Sciences, Uppsala, Sweden; ^2^Swedish Biodiversity Centre, Swedish University of Agricultural Sciences, Uppsala, Sweden; ^3^Department of Clinical Sciences, Swedish University of Agricultural Sciences, Uppsala, Sweden; ^4^World Organisation for Animal Health, Standards Department, Paris, France; ^5^Department of Animal Science and Large Animal Clinical Sciences, Michigan State University, East Lansing, MI, United States; ^6^Department of Sustainable Agricultural Systems, University of Natural Resources and Life Sciences, Vienna, Austria

**Keywords:** animal well-being, sustainability, conflicts, synergies, one welfare, animal welfare, sustainable development goals

## Abstract

This paper systematically evaluates the extent to which achieving the UN sustainable development goals (SDGs) is compatible with improving animal welfare. The analyses were based on discussion and independent scoring in a group of 12 participants with academic backgrounds within agricultural or veterinary sciences. We considered all categories of animals; those kept for food production, working and companion animals, but also laboratory and wild animals. The strengths of the links between improving animal welfare and achieving an SDG were scored on a 7-point scale, from being completely indivisible, at one end of the scale, to where it is impossible to reach both the SDG and improved animal welfare at the same time. There was good consensus between participants, with the overall scores being positive, indicating that although animal welfare is not explicitly mentioned in the SDGs, working to achieving the SDGs is compatible with working to improve animal welfare. When analyzing the direction of the links, the impact of achieving an SDG was considered, on average, to be slightly better at leading to improved animal welfare, than the impact of improving animal welfare was on achieving the SDG. The exception to this was for SDG 2, dealing with zero hunger. The two SDGs for which there was strongest mutual reinforcing were SDG 12, which deals with responsible production and consumption, and SDG 14, which deals with life below water. Most of the targets under these two SDGs were considered relevant to animal welfare, whereas when all SDGs were considered, 66 targets of the total of 169 were considered relevant. Although the results of this study suggest a mutually beneficial relationship between improving animal welfare and achieving SDGs, this should be confirmed on a wider group of people, for example people from less developed countries and other stakeholders. Showing the relationships between animal welfare and the sustainable development goals helps highlight the importance of animal welfare when implementing these goals in practice. The methodology described in this study could also be useful to researchers working with other societal and environmental issues not yet considered within the overall SDG framework.

## Introduction

In 2015, the United Nations adopted a set of goals that imagines a future just 15 years off (2030) without poverty and hunger, and safe from the worst effects of climate change and loss of biodiversity (1). These Sustainable Development Goals (SDGs) have a wide scope, but the role of our domesticated animals as well as wild animals, including fish, is hardly mentioned and their welfare is not mentioned at all.

The most widely used definition of sustainable development is the one proposed by the UN World Commission on Environment and Development in its report Our Common Future (2): “Sustainable development is development that meets the needs of the present without compromising the ability of future generations to meet their own needs.” Another focused on sustainable livestock systems is: “A system or procedure is sustainable if it is acceptable now and if its effects will be acceptable in future, in particular in relation to resource availability, consequences of functioning and morality of action” (3). Sustainable development aims to balance different needs toward achieving dignity, peace and prosperity for people, against an awareness of the environmental, social and economic limitations we face as a society. This implies a holistic approach, fully considering the wider and future impacts of different, and often competing, needs. Holling (4), who discusses the complexity of the issues declares that sustainability can be seen as the capacity to create, test, and maintain adaptive capability and that the phrase “sustainable development” refers to the goal of fostering adaptive capabilities and creating opportunities.

The 193 member states of the UN agreed on 17 SDGs covering central principles of reducing poverty and hunger, improving health and well-being, and creating sustainable production and consumption patterns. It is an ambitious plan and one can discuss whether the 17 SDGs are the “right” ones, formulated in the “best” way, but they form the only globally agreed common framework for people and the planet (5). Under each SDG a series of targets is formulated as well as indicators to monitor progress toward the targets. There are currently 169 targets and there is a complex network of interactions between them. It is important to understand these interactions in order to align activities toward balanced outcomes. Methodologies are starting to be developed to assess interactions between targets and to explore how they might be visualized (6, 7). This is both conceptually and practically challenging.

It is almost inevitable, given the complexity of the task, that not all relevant areas and aspects are explicitly covered by the SDGs. The contribution of animals in achieving the SDGs is not recognized nor made explicit. Nevertheless, there are obvious areas where animals play an important role in the context of sustainable development. These include for instance food security, transport, employment, and livelihoods. There are less positive effects of man's interaction with animals also, as well as a number of drawbacks associated with continuous growth and intensification of the animal sector. These include challenges to the environment (gaseous emissions, water and soil pollution, and ecosystem damage), issues regarding animal welfare (animal abuse and negative consequences of intensive selection and production), and animal and human health (zoonotic diseases and inappropriate use of antimicrobials and anthelmintics).

The relevance of good animal welfare and health for sustainable development is acknowledged elsewhere e.g., the Food and Agriculture Organization (FAO), the World Organization for Animal Health (OIE), and the World Health Organization (WHO) agreed in 2010 to share responsibilities and coordinate global activities to address health risks at the animal-human-ecosystems interfaces (8). More recently the UN Committee on World Food Security proposed draft recommendations on sustainable agricultural development for food security and nutrition including the role of livestock (9). Recommendation “D” of Article VIII, entitled “Animal health and welfare” reads: “Improve animal welfare delivering on the five freedoms and related OIE standards and principles, including through capacity building programs, and supporting voluntary actions in the livestock sector to improve animal welfare.” This was the first time in the UN's 71 year history that animal welfare had been identified as a global goal of sustainable agricultural policy (10). In light of these advances in sustainable agriculture policy, there is an underlying premise that there exists a universal definition of animal welfare.

Animal welfare is the physical and mental state of an animal in relation to the conditions in which it lives and dies. An animal experiences good welfare if the animal is healthy, comfortable, well-nourished, safe, is not suffering from unpleasant states such as pain, fear and distress, and is able to express behaviors that are important for its physical and mental state. Good animal welfare requires disease prevention and appropriate veterinary care, shelter, management and nutrition, a stimulating and safe environment, humane handling and humane slaughter or killing. While animal welfare refers to the state of the animal, the treatment that an animal receives is covered by other terms such as animal care, animal husbandry, and humane slaughter/killing (11).

Today, protecting the welfare of animals has unequivocally entered the public policy mainstream in a growing number of countries, with significant public and private regulations governing the welfare of animals in our care (10). In many countries this not only relates to production animals but also to sport and companion animals, laboratory animals or those used in animal assisted therapy. Animal welfare science has become a well-established discipline in its own right, greatly extending our understanding of positive as well as negative animal physiological and psychological states and our means to appropriately respond to them within the practices of animal production and of human/animal interactions in general.

Increasingly, the interconnections between animal health and welfare, and human health and welfare as well as their relation with environmental factors (climate change, biodiversity) are being recognized, as shown by the emergence of the “One Welfare” concept (12). One Welfare extends and complements the One Health theme used for human, animal, and environmental health (13, 14). There are obvious parallels between One Welfare, One Health themes, and the SDGs, but as yet these are not clearly defined nor are efforts within these different areas coordinated to utilize any synergies.

**Figure 1:**
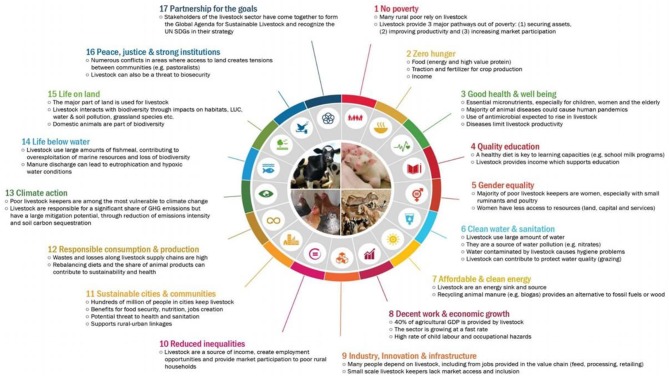
Figure developed by the Food and Agricultural Organization of the United Nations (15) on how animal production can contribute to the different sustainable development goals (SDGs).

Animal health and welfare are closely linked to animal productivity. Good animal welfare has therefore a direct and indirect beneficial financial impact, helps to reduce poverty and has gender implications, as often women care for livestock (see Figure 1 for other examples). But of course including animal welfare in sustainable development is more than developing sustainable livestock production systems. Animal welfare is a common good and, as such, a shared responsibility and an ethical obligation. A common good is typically achieved through actions of a community that result in uplifting the well-being of its members. It can be manifested through a sense of shared values such as the welfare of animals. A common good differs from a public good. A public good has two primary traits, the first is non-excludability meaning it is there for all to use and no one can be excluded. The second is non-rivalry, which means a person's consumption of that good does not diminish another person's ability to access it. Typically, public goods are available through government action and financed through public funds (16). Nevertheless, it is no easy task to integrate targets for the many different categories of animals (food producing, working, laboratory, pet, sport, and wild animals) and the different ways in which we interact with them in a balanced way into the various SDGs.

There have been a vast number of studies published about the SDGs describing what they cover, what has been neglected in them or how they should more easily be met. For instance, the UN Permanent Forum on Indigenous Issues has found that 73 of the 169 targets within the SDG have strong links to the UN Declaration of the Rights of Indigenous Peoples and they have consequently been working systematically within the SDG framework to ensure that indigenous peoples are not left behind in the 2030 agenda (17). Literature surveys in Web of Science and Google Scholar show an almost complete absence of studies relating SDGs to animal welfare. As explained above, animal welfare is a relevant aspect of sustainable development. Therefore, an understanding of how animal welfare is affecting the SDGs, and vice versa, is essential to formulate balanced targets that take account of animal welfare aspects. This study aimed to pilot a potential methodological approach toward the analysis of this interaction and provide an initial characterization of the relationship based on a panel of experts in animal welfare and environmental issues.

## Methodology

The information in this paper is based on two exercises carried out at a workshop called “Animal Welfare and the Sustainable Development Goals” organized at the Swedish University of Agricultural Sciences in June 2018 as part of the Global Challenges University Alliance (GCUA) workshop series and one follow-up home exercise. The 12 active participants were from 8 countries (Sweden, USA, Chile, Italy, Germany, UK, France and Mexico). They worked at a university that was part of the GCUA and had an interest in this topic, or were an invited speaker. Participants therefore responded to the opportunity to attend and were not selected to be a representative sample. They were mainly academics (six veterinarians, three animal scientists, two biologists and an ecologist) from the general areas of animal welfare, sustainability and biodiversity. There were seven females and five males.

The methodological approach consisted of three steps.

1. **Mapping Potential Links Between Animal Welfare and Each SDG**
  Before attending the workshop people were asked to think about potential areas in each of the 17 SDGs where there could be links to animal welfare. Each participant was also allocated 3 SDGs for which they were asked to consider this in more detail. The outcomes of this preliminary independent pre-workshop assignment were discussed at the workshop and a list of areas in which there are potential links between animal welfare and each specific SDG were identified in a brain storming session. We considered all categories of animals, domesticated and wild, and aimed to consider all interactions between animal welfare and the SDGs for them, in high income as well as low-income countries.
2. **Scoring the Strength of the Link Between Animal Welfare and Each SDG and the Direction of the Link**
  The group then discussed whether or not to select specific SDGs and their relationship to animal welfare for further investigation. Considerations were the number and diversity of links between that particular SDG and animal welfare and their potential strength and importance. Since context and time scale matter for such scoring, it was also discussed if using the single goal of “improved animal welfare” and linking it to each of the 17 different SDGs was the optimal approach. Other ways of defining animal welfare, perhaps splitting it into several goals addressing different dimensions of animal welfare, were therefore discussed, but in the end not applied.
  The outcome of the discussion about how best to define the goal of animal welfare was to use the single entity of “improved animal welfare” and link to the main text of the SDG. For convenience we used the phrase proposed by the World Animal Health Organization “Animal welfare means the physical and mental state of an animal in relation to the conditions in which it lives and dies” as the definition of animal welfare in this exercise (11). However, we did decide to score the link separately for the two directions.
  Using the scoring system developed by Nilsson et al. (6) the links were scored on a 7-point scale from indivisible (score +3: where the successful achievement of the SDG is inextricably linked to improved animal welfare), to canceling (score−3, where it is impossible to reach both the SDG and improved animal welfare at the same time). These are presented in Table 1. Participants independently scored the links between each SDG and animal welfare using an electronic scoring software (MENTIMETER®, Version 2.0.4.2018). The scoring was performed for both directions of the link; animal welfare impact on SDGs and SDGs' impact on animal welfare. Taking SDG 1 as the example, the task was firstly “to score the consequence of ending poverty on animal welfare improvement” and secondly “to score the contribution of improving animal welfare on ending poverty.” This was then repeated for all 17 SDGs resulting in 34 scores per individual, with the exception of one person who missed answering one question.
  An initial descriptive analysis was done with the scores obtained at the goal level. The sum of scores was determined as well as the mean score and range for each SDG vs. animal welfare, taking into account the direction of the assessment. Results were plotted (scatter plot with weights and radar-plot) to attain a better qualitative understanding of the associations as valued by the panel of experts. To evaluate if there was a score difference in the impact of achieving a specific SDG on animal welfare vs. the impact of improving animal welfare on the achievement of an SDG, a Wilcoxon-signed-rank test was done among the pair of scores given by the 12 participants. All analyses were done using SAS® software (version 9.4).
3. **Qualitative Exploration of Links Between Animal Welfare and the Targets Under SDGs**
  After the meeting, participants were asked to consider in more detail the targets under each SDG and their links to animal welfare. Pre-workshop the participants had done this only for the three allocated SDGs. Given that there are 169 targets, a full scoring was considered impractical at this stage. Rather participants were asked to decide whether each target was associated with animal welfare or not. The number of targets associated with animal welfare was then counted.

**Table 1:** The scoring system used to rate the strength of the links between improving animal welfare (AW) and achieving a particular sustainable development goal (SDG).

**Interaction**	**Name**	**Explanation**
+3	Indivisible	AW and the SDG are inextricably linked to each other, so that achieving one results in achieving the other.
+2	Reinforcing	Improving AW aids the achievement of the SDG or, alternatively, achieving the SDG aids improving AW.
+1	Enabling	Improving AW creates conditions that furthers the achievement of the SDG, alternatively, achieving the SDG furthers improving AW.
0	Consistent	No significant interactions between AW and the SDG.
−1	Constraining	Improving AW limits options to achieve the SDG, alternatively, achieving the SDG limits options to improve AW.
−2	Counteracting	Improving AW clashes with achieving the SDG, alternatively, achieving the SDG clashes with improving AW.
−3	Canceling	Improving AW makes it impossible to achieve the SDG, alternatively, achieving the SDG makes it impossible to improve AW.

*Adapted from Nilsson et al. (6)*.

## Results

### Mapping Potential Links Between Animal Welfare and Each SDG

The results of the brainstorming session to propose potential links are summarized in Table 2. The majority of the identified links were related to animals kept for food production (farm or aquaculture) or to working animals (equids), but in some cases the links could apply to all categories of animal. An example is in SDG 1, where allied industries providing services to animal owners would benefit from reduced poverty. This would apply to owners of any category of animal, not only farmers. Another example is in SDG 4, as education for children can relate to animals in general. Some links were nevertheless specifically associated with companion animal welfare or wildlife. In SDG 3 owning a pet was mentioned as being associated with improved physical and psychological health of the owner. Whereas, in SDG 11 an example was given about the importance of urban wildlife management and in SDG 14 several examples related to wild fish. The welfare aspects mentioned in connection with the SDGs were almost exclusively related to animal health and productivity. Although there did seem to be variation between SDGs in the number of links to animal welfare, and in the consequences of the link for sustainable development or for animal welfare, links were identified for all SDGs and so it was decided to continue to investigate all SDGs in the next stage.

**Table 2:** Outcome from brainstorming exercise on the links between animal welfare and the sustainable development goals (SDG).

**SDG**	**Links between animal welfare and the sustainable development goals**
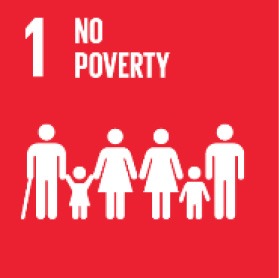	• Improved welfare of farm animals may provide paths out of poverty via increased productivity and production efficiency, decreased veterinary care costs, lengthening of the production life, increased fertility, increased product quality or value, and access to new markets. • Allied industries, i.e., those providing services to animal owners, may also benefit from reduced poverty. • In the case of working animals (e.g., equids), improved welfare contributes to increasing transport and carrying capacity, so promoting income.
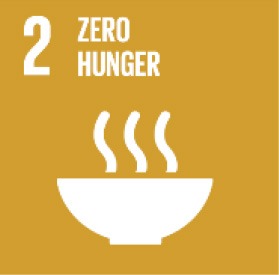	• Improved welfare of food animals leads to more meat, milk and eggs, and also to improved product quality, so decreasing food losses and wastes. • In the case of working animals it contributes to increasing agricultural production. • Maintaining genetic diversity may contribute to maintaining good animal health and welfare sometime in the future. • Biodiversity (e.g., pollinating insect populations) may promote better grazing opportunities with a wider range of plants for animals on pasture, leading to better nutrient recycling and hence improved meat and milk production. • Improved nutritional status of animals may come at the cost of increased hunger in human populations because of food-feed competition.
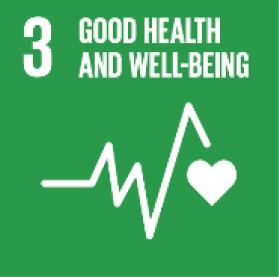	• Good welfare in animals increases their immuno-competence and resistance to zoonotic diseases, that can be transferred to humans, allowing decreases in the use of antimicrobials, and so reducing the risk for multi resistance. • Owning a pet can be associated with improved physical and psychological health. Animal-assisted therapy is used for various physical and psychological disorders, so contributing to human well-being.
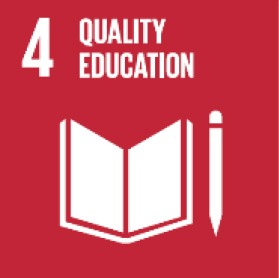	• Educating children about animals can improve empathy and reduce interpersonal violence. • Children are the next generation of consumers who can create a market for enhanced welfare products. • Education of farmers, and those interacting with animals, can change attitudes toward animal welfare and farmers can share knowledge about animal husbandry practices in community based projects. • Provision of information to adults (consumers and citizens) affects societal attitudes and demand related to animal production, as well as how pet and sports animals are treated.
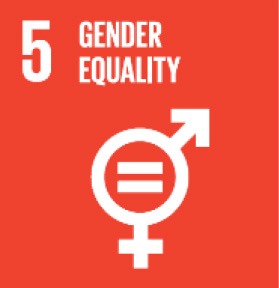	• Animals are often cared for by women and improving the status and welfare of animals enhances their role. • Improving the welfare of animals in a community also improves empathy between different groups within their societies and reduces violence among genders.
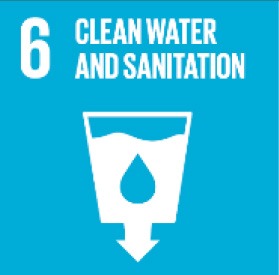	• Clean water and sanitation are important for the health of both animals and humans, so there are mutual benefits. • In times of shortage, competition for water may be a problem. Animals may also contaminate drinking water.
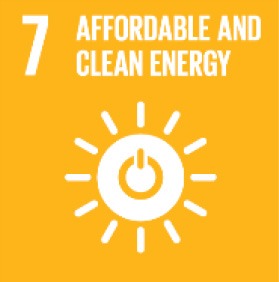	• Animals or their waste products can be used to create renewable energy, increasing their importance and value to the community. • Increasing the welfare of draft animals improves their performance, so providing an improved energy source and simultaneously increasing animal welfare.
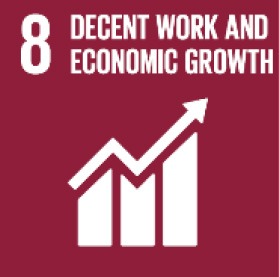	• Sustainable livestock systems developed for a specific region can increase the economic value of the animals leading to additional incentives to improve welfare and vice versa. • Economic growth and incentives in the short term can make it possible for farmers to leave systems where animal welfare is substandard. • Links to animal welfare incentives can improve worker job satisfaction e.g., in slaughterhouses. • Appropriate animal handling, adapted to the nature and behavior of the animals, reduces animal's stress as well as risks and occupational hazards for workers. • Working with animals or having pets at the work place can also enhance the working environment. • Working dogs (drug control, dogs for the blind etc.) work better when their welfare is good.
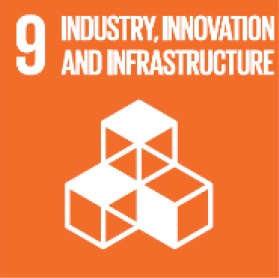	• There are business opportunities to develop new systems and technologies that also enhance animal welfare. Interest in the welfare of farm, companion, laboratory animals etc. can lead to new industries to supply this demand and to new innovation opportunities.
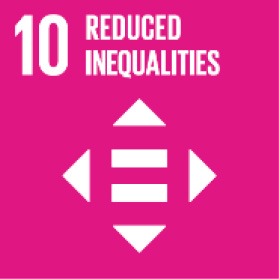	• Harmonization of animal welfare standards globally reduces inequalities and provides possibilities for increased trade of high animal welfare products as well as preventing trade inequalities leaving some countries behind. • Financial loans to industries as well as those to small-holder farmers can be conditional on improved animal welfare. • Sharing of veterinary services (PVS pathway) can reduce inequalities in animal disease control.
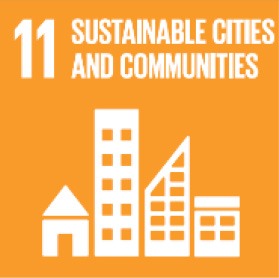	• Having farm animals near cities can improve possibilities for education about animals as well as improve food security and reduce the distances live animals are transported. • Cities can be designed to be pet friendly (e.g., dog parks) and responsible ownership reduces stray dogs with its associated human health aspects. • Urban wildlife management and reducing habitat loss improves biodiversity and sustainability, but also requires that waste production from cities is managed appropriately.
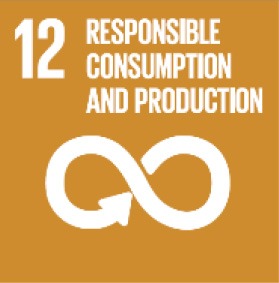	• The responsible and restrictive use of antimicrobials requires good animal welfare, but also minimizes development of antimicrobial resistance. • Changing our consumption patterns in order to use the entire animal more efficiently, will reduce environmental load and reduce the number of animal lives used in total. • Feeding ruminants only with feed that is unsuitable for humans avoids competition over certain food sources and improves sustainability. • Decreasing consumption of food of animal origin (which includes fish) and increasing the willingness to pay the true cost of animal-derived food will increase the possibility for farmers to improve the welfare of the animals they keep and reduce the negative environmental consequences of high animal protein diets.
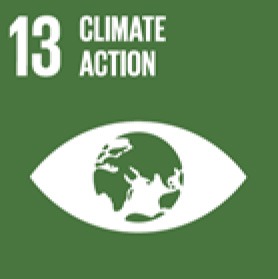	• Climate change increases the risk that animals will be exposed to new diseases. Animal species should be kept or used for farming in the climate in which they evolved or where the breed was selected. • Although there are many uncertainties when calculating the carbon footprint of livestock products, it is general agreed that there is a link to animal welfare in that production efficiency and longevity are improved in animals with good welfare.
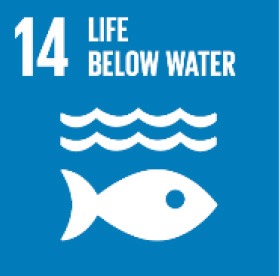	• Improved welfare of farmed fish leads to a reduction in the need for antibiotics in aquaculture. • There are synergies e.g., reducing plastics benefits both fish welfare and the environment. • Improved methods of catching wild fish will improve their welfare, the quality of wild fish product and reduce by-catch. • Creating a demand for alternative fish species will reduce the wastage associated with by-catch and may reduce demand for threatened species. • Appropriate selection of fish for aquaculture, better adapted to the environmental conditions, will improve fish welfare and the sustainability of the production generally.
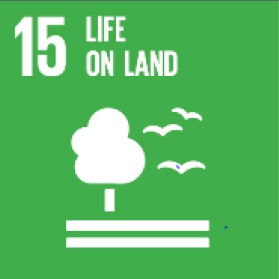	• Modified approaches to grazing can reduce soil loss, improve carbon sequestration, and increase the diversity of soil biota. • A well-balanced grazing on meadows or semi-natural grasslands contributes to biodiversity • Providing people with farmed sources of protein, produced according to good animal welfare standards, will reduce illegal hunting, illegal trade, and reduce the risk of transmission of zoonoses. • Responsible ownership of animals (farm and pets) can reduce the incidence of detrimental interactions with wildlife.
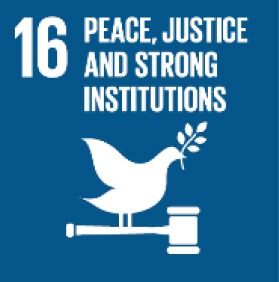	• Improved governance of veterinary services and competent authorities can guide and enforce good animal welfare policies. • Increased participatory and representative decision making, such as by stakeholder involvement, will help ensure that animal welfare regulations are appropriate and enforceable. • Animal welfare is at risk where governance is functioning poorly or in countries at war.
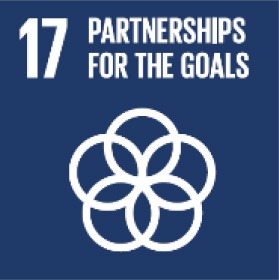	• Public private partnerships can be effective nationally and globally in supporting initiatives to improve animal welfare. • Trade agreements can support welfare developments, providing financial support and incentives to improve animal welfare. • Providing support for countries to reduce their national debt and lift their possibilities to develop their domestic capacity may indirectly also improve animal welfare according to many of the links identified in earlier goals.

### Scoring the Strength of the Link Between Animal Welfare and Each SDG and the Direction of the Link

**Figure 2:**
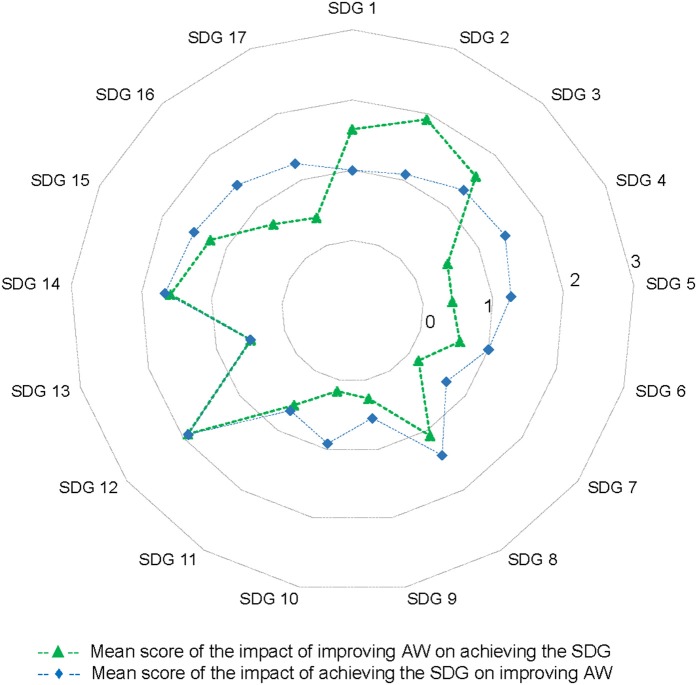
Overview of the average score for each sustainable development goal (SDG)—animal welfare (AW) link. The green line (dashed line with triangles) refers to the impact of AW on achieving the SDG and the blue line (dotted line with squares) to the impact of the SDG on achieving improved AW.

The overall mean score was positive, showing an overall co-benefit between achieving sustainable development and improving animal welfare. The average score for the impact of achieving the SDG on improving animal welfare was slightly stronger (1.15) than the effect of improving animal welfare on achieving the SDGs (0.89). However, this was not consistent across all SDGs, as can be seen from the radar-plot (Figure 2). For example, for SDG 2 participants scored that the impact of improved animal welfare on ending hunger was stronger than the effect of achieving the SDG on improving animal welfare (W = 2.59, *p* = 0.047). Whereas, for SDGs 4, 5, 10, 16, and 17 participants scored that the impact of achieving the SDG on improving animal welfare was stronger than the impact of improved animal welfare on enabling the SDG (W = −18, *P* = 0.008; W = −18, *P* = 0.008; W = −14, *P* = 0.002; W = −18, *P* = 0.008; W = −22.5, *P* = 0.004, respectively). For the other SDGs the scores in the two directions did not differ.

**Figure 3:**
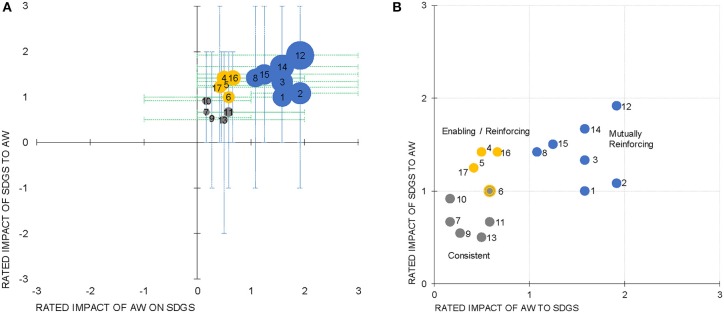
**(A)** Scatter plot of the rated impact of the sustainable development goals (SDGs) on animal welfare (AW) (Y-axis) vs. rated impact of improving AW on the SDGs (X-axis). The size of the bubble represents the total sum of the scores given by the participants. The bars show the minimum and maximum score given by the participants. **(B)** Scatter plot focused on the top right quadrant of (A). Y-axis is the impact of the SDG on AW. X-axis is the impact of improved AW on the SDG. Three main groups of SDGs are marked: (1) the consistent group (gray dots), (2) the enabling/reinforcing group of SDGs (yellow points), and (3) the mutually reinforcing group (blue).

The mean score and range of scores from participants were plotted on two axes, one showing the impact of achieving the SDG on animal welfare and the other the impact of improving animal welfare on the SDG (Figure 3A). Noticeable from this Figure is the grouping of all means in the top right quadrant of the axes, reflecting the mainly positive scores. Additionally, from this Figure we can see the differences in the range around each SDG. The minimum range (most consensus that there was no or little association) was for SDG 9 which deals with industry innovation and infrastructure (effect of improved animal welfare on SDG ranged from 0 to 1) and SDG 7 which deals with affordable clean energy (effect of achieving the SDG on animal welfare ranged from 0 to 1). On the other hand, the maximum range was for the effect of SDG 13 (range −2 to 2) and SDG 8 (range −1 to 3) on animal welfare; where SDG 8 deals with decent work for all and economic growth, and SDG 13 deals with climate action.

Taking the average of the lowest scores (most negative in the range) of answers for each of the 34 questions, or the average of the highest scores (most positive) for the questions, allows us to generate a worst-case and a best-case scenario for the links between animal welfare and the SDGs. However, even taking the worst-case scenario (Figure 3A), the average score for improving animal welfare on the SDGs is −0.11 and for the impact of achieving the SDGs on animal welfare the mean score is −0.3. This score of around zero would be in the consistent range, having no significant positive or negative interactions (Table 1). Taking the best-case scenario (Figure 3A), where the highest scores for each of the questions are considered, the mean score for improving animal welfare on the SDGs is 2.2 and, for the impact of achieving the SDGs on animal welfare, the mean score is 2.5. This score is in the reinforcing range, meaning that it aids the achievement of another goal (Table 1).

Most interesting is, what this (Figure 3A) tells us about the different SDGs and their relationship to animal welfare. Figure 3B zooms in on the top right quadrant and it can be seen that the SDGs can be separated into three groups. There is a high mutual enabling of the SDG on animal welfare and of improved animal welfare enabling the SDG, for SDGs 1, 2, 3, 8, 12, 14, 15. Those SDGs where there is a mutually consistent relationship are SDGs (6), 7, 9, 10, 11, and 13. SDG 6 is on the border between groups. Therefore, for 13 of the 17 SDGs there was a relatively symmetrical relationship between the two directions of the link. An asymmetric relation (differences in the strength of the link according to the direction) occurred only in the situation where there was an *enabling* of the impact of improving animal welfare on achieving the SDG, and a *reinforcing* of the impact of achieving the SDG on improving animal welfare. These “enabling/reinforcing” SDGs were 4, 5, (6), 16 and 17. There were no SDGs with the equivalent, but opposite symmetric relation, although SDG 1 is borderline (Figure 3B).

### Qualitative Exploration of Links Between Animal Welfare and the Targets Under SDGs

Given that from the scoring in the workshop, all SDGs were found to be linked to animal welfare, it is not surprising that at least one of the targets under each SDG, but often several, could also be linked to animal welfare in the follow-up home exercise. For some SDGs e.g., SDG 1, 2, and 12 many of the targets were linked to animal welfare, whereas for others a much smaller proportion of the targets were considered relevant. In total 66 out of the 169 targets were considered relevant to animal welfare by all four participants who completed this task.

## Discussion

The two most noticeable outcomes of this series of exploratory exercises on the relationships between achieving the SDG and improving animal welfare are, firstly, the considerable consensus in scoring between participants and, secondly, that of the 34 relationships scored (17 SDGs × 2 directions of influence) 29 were on average positive, and none were on average negative. That is to say, in the opinion of the participants there is no conflict between achieving an SDG and improving animal welfare, rather creating the one actually helps achieving the other. In the following sections we discuss these results and make suggestions for further research in this new area.

### Relationships Between the SDGs and Animal Welfare

The first point to be discussed is the reliability and the generalizability of these views. The sample size of respondents was small and most people were employed by a university, so the participants were clearly not a representative sample. The study should be repeated with a wider range of stakeholder categories and with a broader demographic sample to see whether similar scores are obtained. Although all participants were interested enough in the general area to attend the workshop, they had different backgrounds and came from different regions in Europe and the Americas, even if these countries are similar in terms of economic development. Knowledge of sustainable development and animal welfare, both of which are complex areas combining science, practice, and ethical aspects, also varied. It can also be assumed that the participants' view on the multidimensional concept of animal welfare varied, i.e., whether emphasis is more put on biological functioning, the emotional well-being or the so-called naturalness (18). The statements in the initial mapping of potential links (Table 2), however, primarily addressed aspects such as the health or nutritional state of the animals, indicating that the biological functioning approach was mostly considered in relation to animal welfare and SDGs. We did not ask people to specify which species, or which activities involving animals they were thinking of when they made the scoring. We tentatively speculate that the assessments made in this exercise will be biased toward the normative values (19) of the participants and touch on the wicked problems (20) that can arise from this source of social complexity (21).

The range of scores for a particular correlation was also never more than four score points, out of the potential seven, which furthermore supports good consensus, even if we cannot exclude the risk for a positive bias in the scoring because of initial interest in the topic. We discuss the findings of these exercises, but put most emphasis in this discussion on the questions they raise for further research and on the methodological aspects.

### Symmetry and Asymmetry in the Links

From the scoring it seems to be that achieving the SDGs are in general a stronger enabler for improving animal welfare than the other way around. Given the breadth of each of the SDGs, this is perhaps not surprising so we instead ask the question—Why this was *not* the case for SDGs 1 and 2 (although only significant for SDG 2), dealing with no poverty and zero hunger? Some indications of why animal welfare may be a stronger enabler for achieving these SDGs came out of the brain storming, summarized in Table 2. These goals can be clearly linked to the production of food, much of which comes from animal sources, or involve working animals to help in the production. Thus, improving animal welfare can contribute to achieving improvements in animal and food production and thus a way out of poverty and hunger.

The clearest asymmetry in the opposite direction, when achieving the SDG has a greater impact on improving animal welfare, was for SDGs 4, 5, 10, 16, and 17, dealing with education, gender equality, reduced inequality generally, justice, and partnerships. While there are arguments, as illustrated by FAO (Figure 1), in how promoting animal welfare and improved livestock production can help achieve these SDGs, the participants in the workshop regarded the link to be strongest in the reverse direction. These particular goals are related to a better functioning and more equal society. It could be suggested that such a society facilitates increasing awareness of the importance of animal welfare or providing the mechanisms for it to be improved. There is, for example, increasing awareness of the importance of educating children about animals, to prepare them for their role as future consumers (22, 23), and gender equality promotes the role of women who are shown to give higher importance to animal welfare than men (24).

While acknowledging that educating children as future consumers will inevitably link to livestock production, irrespective of the level of development of the country, SDGs 4, 5, 10, 16, and 17 are also very relevant to other categories of animals. Education is just as likely to involve companion animals and enforcement of legislation is just as likely to be related to laboratory animals or to conservation issues. In summary, including other categories of animals and thinking globally leads to even more links between animal welfare and sustainable development, as already demonstrated in the One Health and One Welfare approaches (12–14).

### Focusing on the Mutually Enabling SDGs

While there was clearly an overall positive relationship between achieving an SDG and improving animal welfare, the link reached the *reinforcing* level only for some SDGs. The SDGs 12 and 14 were strongest in this respect, in that their average scores were approaching 2. These SDGs deal with responsible consumption and production, and life below water, respectively. The scores are perhaps not surprising given the long established discussions on sustainable agriculture and fishing practices (25).

In Figure 3B, we classified all those SDGs which had an average score in both directions of >1, as “mutually reinforcing.” It is interesting to see if there are any commonalities between these “mutually reinforcing” SDGs and if these, in some way, contrast with communalities of those SDGs which had an average score of <1, which we have called “consistent.” A third group was called “enabling/reinforcing.” Perhaps the clearest difference is that both the “mutually reinforcing” and “enabling/reinforcing” groups (blue and yellow in Figure 3B) are anthropocentric or zoocentric SDGs, i.e., dealing with humans or other living beings. On the other hand, the “consistent” SDGs deal with more “technical” issues, like affordable and clean energy, industry and innovation, trade inequality, sustainable cities and climate. Although it is interesting that despite their emphasis on industry and urban life, achieving these SDGs was still considered not to be in conflict with achieving improved animal welfare. Reasons for this become apparent from the brain storming exercise summarized in Table 2. They seem to relate to the contribution of agricultural innovation to safeguarding the welfare of animals, for example, by reducing the use of equids for heavy transport in developing countries or by using technologies in the area of precision livestock farming to monitor and improve animal welfare. Furthermore, it is also in the discussion of these “consistent” SDGs that companion animals were mentioned in the brain storming; they play a role in the spread of disease via waste or in problems related to stray dogs in cities.

### Range of Scores Between Participants

The range of scores was smallest among the 12 participants for SDG 7 and SDG 9 (dealing with affordable and clean energy, and industry, innovation, and infrastructure, respectively), and greatest for the effect of SDG 8 and SDG 13 (dealing with decent work for all and economic growth, and climate action, respectively). It is not clear why the minimum and maximum range of scores occurred for just these SDGs, but it has been discussed that context when scoring; the country, geography, and technology level, is important (6). In the exercise by Weitz et al. (7), the authors themselves selected the targets for which they wanted to perform the cross impact analysis and they outlined the context whereas we did not do this. It may have been, for example when scoring the link between SDG 8 and animal welfare, that participants had different countries, species or scenarios in mind when scoring. The scores ranged from +3 (indivisible) to −1 (constraining) although there was nobody that scored zero (consistent) and, although only one example, a bimodal distribution would support that participants were considering different scenarios. In contrast, for SDG 9 the majority of participants scored zero for this question, which may possibly imply a lack of scenarios to guide scoring or, irrespective of the scenario, there is little interaction between animal welfare and this SDG. In future studies, it might be worth asking participants afterwards to indicate how certain they felt about their answer, which is often done in expert elicitation during risk analyses (26).

### Selecting SDGs and Further Study of Targets

Even though the link to animal welfare is interesting for all SDGs, it was not feasible to score links between all 169 targets and animal welfare. Has this pilot study helped us decide which targets to focus upon? One suggestion would be to focus on the two SDGs where the links to animal welfare were strongest, namely SGDs 12 (Responsible consumption and production) and 14 (Life below water). These SDGs have 11 and 10 targets, respectively. It would result in 21 links to be scored if we linked each target only to animal welfare. Another option would be to focus on the 66 targets generally considered to have relevance to animal welfare, and to investigate whether there are synergies or conflicts.

### Methodology and Implications for Future Research

As stated in the introduction, other sectors of society have been active when they perceive that their area of interest has not been sufficiently addressed in the SDGs, but rarely has the gap analysis been systematic.

We based our approach on that used by Weitz et al. (7), but there are some notable differences. The first is that we investigated and scored the link between all 17 SDGs and animal welfare, rather than scoring the links between selected SDG targets. Secondly we did not make our scoring context specific, but left that open for participants. There are potential advantages and disadvantages to this strategy. This open approach with no *a priori* selection of SDGs led to some interesting findings regarding links between animal welfare and SDGs that might not have been apparent otherwise. On the other hand, the lack of context may have led to a wider spread of scores between participants. Even if this was the case, the consensus was considerable, implying that people are capable of integrating many different potential scenarios and contexts to come up with an overall score. In this respect, the brainstorming which served the function of raising awareness of the many different contexts probably helped. It could be interesting in a future study to ask people after scoring, which species and scenarios they had in mind when rating the strength of the link.

## Data Availability Statement

The datasets generated for this study are available on request to the corresponding author.

## Author Contributions

GOA and AW performed the statistical analysis. A first draft of the manuscript was prepared by LK, expanded by HB, HT, GOA, MJ, CB, and AW, then further revised by LS, JS, and CW. All authors approved the final version, attended the workshop, and took part in the discussions and scoring.

### Conflict of Interest

The authors declare that the research was conducted in the absence of any commercial or financial relationships that could be construed as a potential conflict of interest.
